# Isolation of *Mycoplasma anserisalpingitidis* from swan goose (*Anser cygnoides*) in China

**DOI:** 10.1186/s12917-020-02393-5

**Published:** 2020-06-05

**Authors:** Miklós Gyuranecz, Alexa Mitter, Áron B. Kovács, Dénes Grózner, Zsuzsa Kreizinger, Krisztina Bali, Krisztián Bányai, Christopher J. Morrow

**Affiliations:** 1 Institute for Veterinary Medical Research, Centre for Agricultural Research, Hungária körút 21, Budapest, 1143 Hungary; 2 Department of Microbiology and Infectious Diseases, University of Veterinary Medicine, Hungária körút 23-25, Budapest, 1143 Hungary; 3 Faculty of Veterinary and Agricultural Sciences, The University of Melbourne, Melbourne, Victoria 3010 Australia

**Keywords:** Antibiotic, China, Mycoplasma, Swan goose, Phallus inflammation, Venereal disease, Whole genome

## Abstract

**Background:**

*Mycoplasma anserisalpingitidis* causes significant economic losses in the domestic goose (*Anser anser*) industry in Europe. As 95% of the global goose production is in China where the primary species is the swan goose (*Anser cygnoides*), it is crucial to know whether the agent is present in this region of the world.

**Results:**

Purulent cloaca and purulent or necrotic phallus inflammation were observed in affected animals which represented 1–2% of a swan goose breeding flock (75,000 animals) near Guanghzou, China, in September 2019. From twelve sampled animals the cloaca swabs of five birds (three male, two female) were demonstrated to be *M. anserisalpingitidis* positive by PCR and the agent was successfully isolated from the samples of three female geese. Based on whole genome sequence analysis, the examined isolate showed high genetic similarity (84.67%) with the European isolates. The antibiotic susceptibility profiles of two swan goose isolates, determined by microbroth dilution method against 12 antibiotics and an antibiotic combination were also similar to the European domestic goose ones with tylvalosin and tiamulin being the most effective drugs.

**Conclusions:**

To the best of our knowledge this is the first description of *M. anserisalpingitidis* infection in swan goose, thus the study highlights the importance of mycoplasmosis in the goose industry on a global scale.

## Background

Production of geese is very important in many parts of the world. Meat and eggs of waterfowl provide foods with high nutritional quality and unique flavour which is believed to be delicious [1]. Waterfowl are also widely used as a source for down and feathers. In some countries, like France and Hungary, geese also produce foie gras comprising engorged fatty goose liver. In Europe and North America geese products are considered as premium quality food sold at high prices while in the Far-East waterfowl are marketed at relatively low prices being a bulk meat source.

Mycoplasma diseases cause enormous economic losses to the goose industry in Europe [2]. The estimated yearly loss of the Hungarian goose industry (25.8 thousand tonnes per year production) [3] inflicted by mycoplasmosis ranged between 2 and 2.5 million euro in the last decades. Numerous *Mycoplasma* species have been isolated from adult geese in association with reproductive disorders. Mycoplasma infection of geese suffering from salpingitis was first reported by Kosovac and Djurisic [4]. Since then beside *Acholeplasma* species, *M. anseris*, *M. anatis*, *M. cloacale,* unidentified *Mycoplasma* species and most frequently *M. anserisalpingitidis* were identified and associated with reproductive diseases in waterfowl [1, 5–8]. After the first laying period about 15 to 20% of the ganders harbour *M. anserisalpingitidis* in the phallus lymph, cloaca and/or trachea. During the laying periods when the ganders are sexually active and under stress, up to 50 to 100% become clinically diseased showing cloaca and phallus inflammation and testicular atrophy [9]. Less frequently salpingitis and vaginitis are observed in the infected breeders [10]. Fertile egg production also decreases. *M. anserisalpingitidis* can induce lethal pathological changes in the embryos and vertical transmission may also occur. Sometimes airsacculitis and peritonitis are seen, even in young birds. General signs such as changes in thirst, decreased food consumption, body weight loss, weakness, nasal discharge, impaired breathing, conjunctivitis, diarrhoea and nervous signs were also described in the affected waterfowl flocks [1, 11, 12].

The annual goose meat production of the world is over 2.5 million tonnes and it is dominated by China (2.4 million tonnes) [3]. In contrast to Europe where the domestic goose (*Anser anser*) is farmed, in China the swan goose (*Anser cygnoides*) is the primarily breeding species. The aim of our study was to investigate mycoplasmosis in swan goose in China with a focus on the presence of *M. anserisalpingitidis*.

## Results

**Fig. 1:**
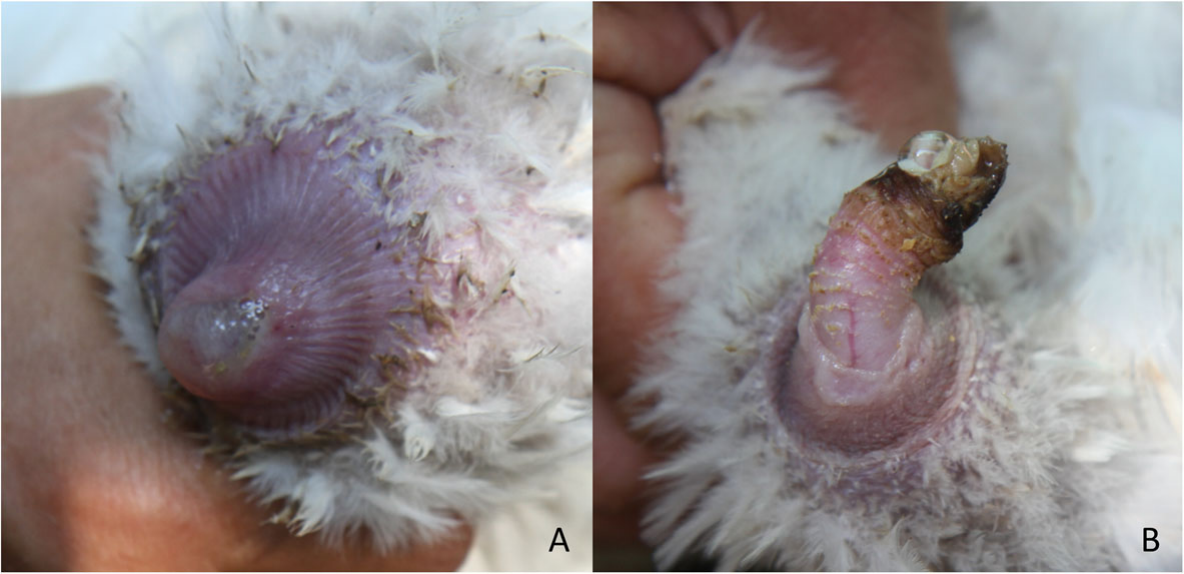
Purulent cloaca (**a**) and necrotic phallus (**b**) inflammation in swan goose

The detailed results of the investigation are summarized in Table 1. Similarly to the European domestic goose, purulent cloaca and purulent or necrotic phallus inflammation were observed in the diseased animals (Fig. 1). According to the owner 1 to 2% of the birds are affected with the disease in each year. *M. anserisalpingitidis* was detected by PCR in the cloaca swab samples of two female and three male birds. The agent was isolated from three geese (three females). From four ganders *M. cloacale* was detected by PCR and was isolated as well. An undetermined *Mycoplasma* species, with 92% 16S–23S sequence similarity to a *Mycoplasma* sp. isolated from a Humboldt penguin in Austria in 2003 (KX863539) was also cultured from one of the ganders.

**Table 1:** Information of sampled swan geese and results of diagnostic examination

SampleID	Gender	Clinical signs	Treatment^a^	PCR	Isolation (Isolate ID)
**1A**	male	cloaca+phallus infl.^b^	TIA + CTC	*M. cloacale*	*M. cloacale* (MYCAV779)
**2A**	male	none	TIA + CTC	*M. cloacale, M. anserisalpingitidis*	*M. cloacale* (MYCAV780)
**3A**	male	cloaca+phallus infl.	TIA + CTC	–	novel *Mycoplasma* sp. (MYCAV936)
**4A**	male	cloaca+phallus infl.	TIA + CTC	–	–
**5A**	male	cloaca+phallus infl.	TIA + CTC	*M. cloacale, M. anserisalpingitidis*	*M. cloacale* (MYCAV781)
**6A**	male	cloaca+phallus infl.	TIA + CTC	–	–
**7A**	male	cloaca+phallus infl.	TIA + CTC	*M. cloacale, M. anserisalpingitidis*	*M. cloacale* (MYCAV782)
**8A**	male	cloaca+phallus infl.	TIA + CTC	–	–
**9A**	female	none	TIA + SPT	*M. anserisalpingitidis*	*M. anserisalpingitidis* (MYCAV783)
**10A**	female	none	TIA + SPT	–	–
**11A**	female	none	not treated	–	*M. anserisalpingitidis* (MYCAV784)
**12A**	female	none	not treated	*M. anserisalpingitidis*	*M. anserisalpingitidis* (MYCAV785)

^a^*TIA* Tiamulin, *CTC* Chlortetracycline, *SPT* spectinomycin^b^*infl.* Inflammation

**Fig. 2:**
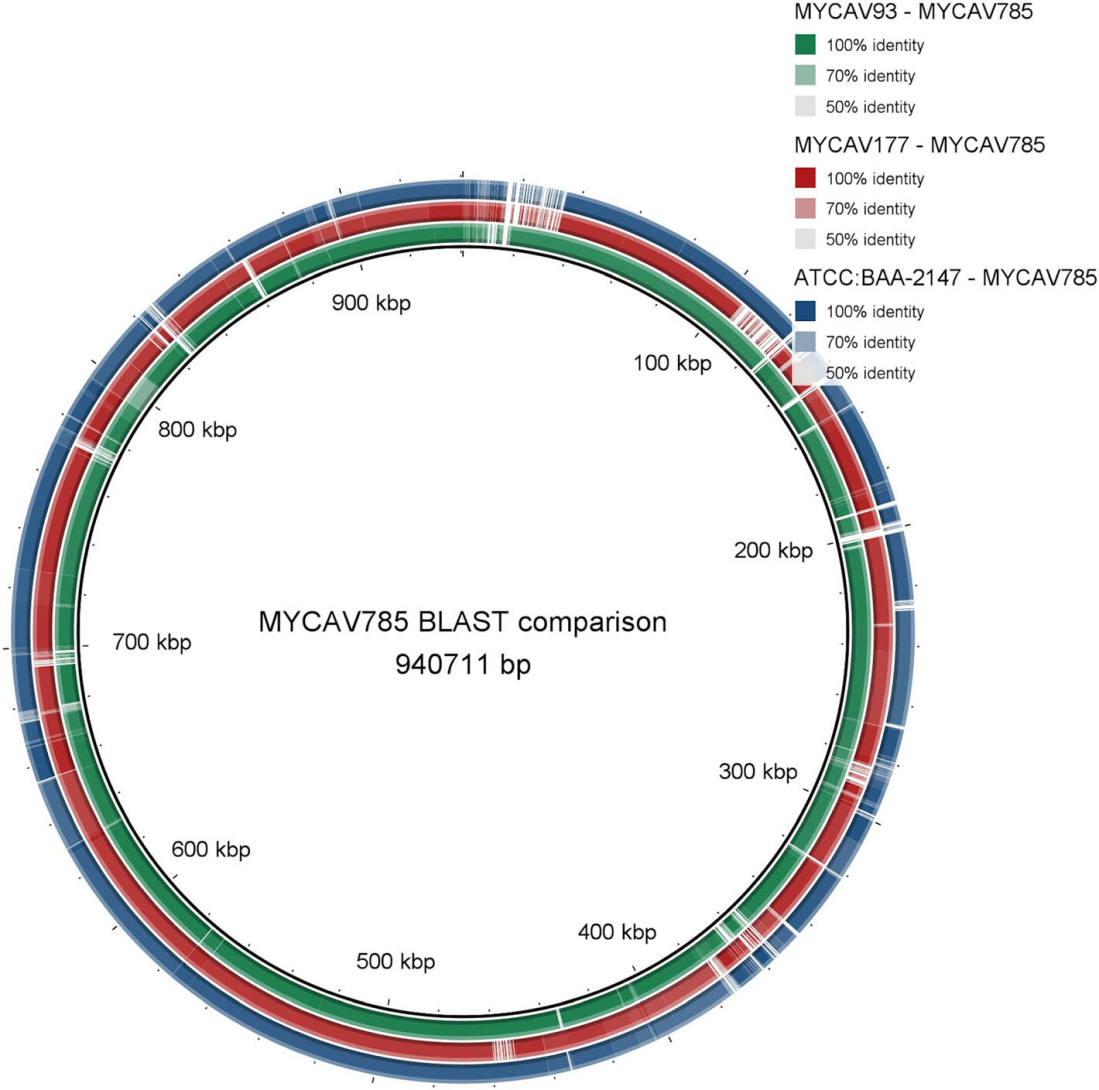
Whole-genome based comparison of *M. anserisalpingitidis* strains from China and Europe. Comparison of the whole genome sequences of strain MYCAV785 isolated from swan goose in China with clinical isolates from domestic goose from Hungary (MYCAV93&177) and the type strain (ATCC BAA-2147). BLAST Ring Image Generator (BRIG) [14]

The sequencing of strain MYCAV785 resulted in more than 5.9 million single reads with the average Phred score over 30 (base call accuracy over 99.9%). The reads have been uploaded to the sequence read archive (SRA) under Bioproject number PRJNA602206. The MAUVE alignment found more than 86,000 single nucleotide polymorphism (SNPs) and over 9000 of these were only present in MYCAV785, approximately 1% of the whole genome of the strain. Based on the BLAST search done with BRIG and the alignment done by MAUVE, MYCAV785 showed highest similarity (84.67% based on MAUVE) with strain MYCAV93, isolated from the inflammated phallus of a domestic goose in Hungary in 2011 [13] (Fig. 2). Although it is important to note that the MYCAV785 genome contains ambiguous nucleotides which may be the reason behind some of the gaps in the BRIG analysis.

**Fig. 3:**
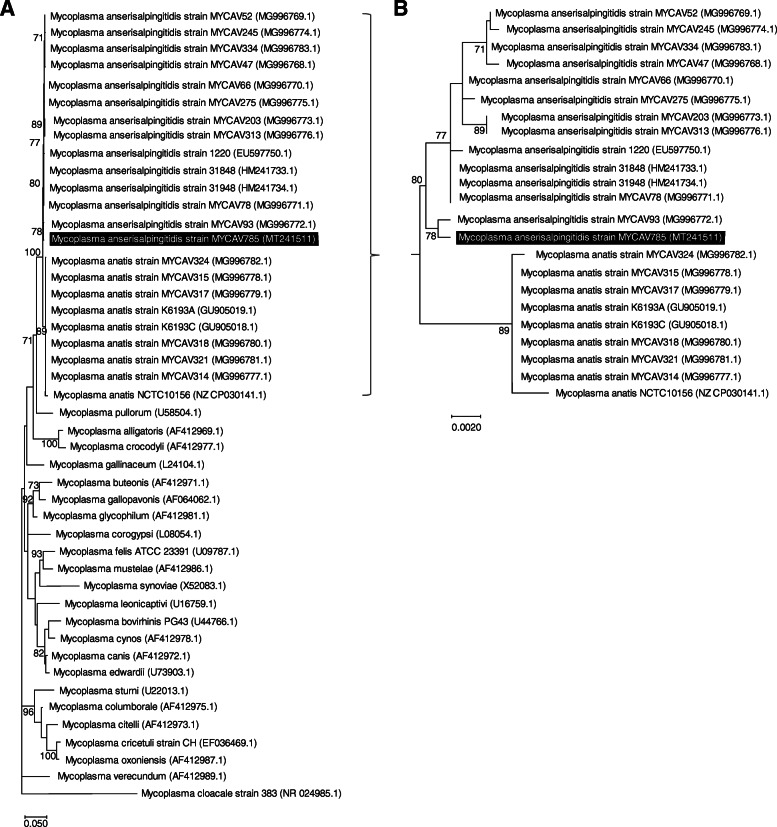
Phylogenetic analysis of the *M. anserisalpingitidis* strain from China within the Synoviae cluster. The dendrograms were assessed using Generalised Time Reversible model with Gamma distribution with invariant sites (GTR + G + I) and 1000 bootstrap in Mega X software [16]. Bootstrap values of ≥70 are shown. Strain MYCAV785, isolated from a swan goose in China is highlighted in black. **a.** Dendrogram based on the nucleotide sequences of 16S rRNA gene of *Mycoplasma* species within the Synoviae cluster. Sequence of the *M. cloacale* was used as outgroup. **b.** Dendrogram based on the nucleotide sequences of 16S rRNA region of *M. anserisalpingitidis* and *M. anatis* strains

The phylogenetic analysis of MYCAV785 based on the 16S rRNA region [15] supported the close relationship between the swan goose isolate and MYCAV93 within the *M. anserisalpingitidis* clade (Fig. 3). Sequence similarity analysis of the *rpoB* gene [8, 17] showed 96.95–97.85% identity between MYCAV785 and *M. anserisalpingitidis* strains available in GenBank, while 90.14–90.24% identity was detected between MYCAV785 and the publicly available *M. anatis* strains. GenBank sequence accession numbers of the 16S rRNA and *rpoB* gene fragments of MYCAV785 are MT241511 and MT241512, respectively (Supplement material).

The *M. anserisalpingitidis* strain MYCAV785, isolated from a non-medicated female goose (12A), showed low MIC values for all tested antibiotics (enrofloxacin, difloxacin, spectinomycin, lincomycin, doxycycline, oxytetracycline, chlortetracycline, tilmicosin, tylosin, tylvalosin, tiamulin, florfenicol and the combination of lincomycin and spectinomycin, at a ratio of 1:2), while isolate MYCAV783 isolated from a treated bird (9A) showed elevated MIC values against all drugs except tylvalosin and tiamulin (Table 2). The growth of the *M. cloacale* strain (MYCAV781) originating from a treated swan goose (5A) was inhibited by low concentrations of the majority of the examined antibiotics, but it showed increased resistance to fluoroquinolones and a slight elevation to tetracyclines and florfenicol.

**Table 2:** Minimum inhibitory concentration values of a *M. cloacale* and two *M. anserisalpingitidis* strains isolated from swan geese

			MIC (μg/ml)												
Isolate ID	Species	Treatment	ENR^a^	DIF	DOX	OTC	CTC	SPT	TYL	TIL	TLV	LIN	LIN-SPT (1:2)	TIA	FLO
MYCAV781	*M. cloacale*	TIA-CTC	2.5	5	0.625	2	4	2	≤0.25	≤0.25	≤0.25	≤0.25	≤0.25	≤0.039	4
MYCAV783	*M. anserisalpingitidis*	TIA-SPT	10	10	10	64	64	8	8	64	≤0.25	> 64	16	0.625	4
MYCAV785	*M. anserisalpingitidis*	not treated	1.25	0.625	0.078	4	2	2	≤0.25	4	≤0.25	≤0.25	0.5	≤0.039	0.5

^*a*^*ENR* Enrofloxacin, *DIF* Difloxacin, *DOX* Doxycycline, *OTC* Oxytetracycline, *CTC* Chlortetracycline, *SPT* Spectinomycin, *TYL* Tylosin, *TIL* Tilmicosin, *TLV* Tylvalosin, *LIN* Lincomycin, *TIA* Tiamulin *FLO* Florfenicol

## Discussion

*M. anserisalpingitidis* is one of the most important disease agent of domestic goose causing significant economic losses of the industry in Europe. It was unknown if this disease agent was present in China where 95% of the global goose production is made using a different species, the swan goose. In previous Chinese publications studying venereal disease in geese the lesions were either correlated with *Escherichia coli* infection, or when *Mycoplasma* isolation was performed as well only unidentified *Mycoplasma*-like cultures were determined [18, 19].

Thus to the best of our knowledge this is the first report describing *M. anserisalpingitidis* infection in swan goose. Similar to the domestic goose, cloaca and phallus inflammation were observed in the diseased birds in China. *M. anserisalpingitidis* was detected and/or isolated from the cloaca swabs of the sampled clinically ill and healthy swan geese like in domestic goose in Europe [20, 21].

The draft whole genome based genetic characterization of the isolated strain showed high similarity with Hungarian *M. anserisalpingitidis* isolates (GenBank accession numbers: CP042295.1, CP041663.1 and CP041664.1) which is interesting considering the geographic distance and different host species. The *rpoB* gene sequence similarity was suggested to be used for interspecies discrimination before, using the cut off value ≥96% [8, 17]. The *rpoB* gene sequence comparison confirmed that MYCAV785 isolate belonged to the species *M. anserisalpingitidis.* The whole genome comparison, and the 16S rRNA and *rpoB* genes based analyses revealed that MYCAV785 showed closest relationship with the domestic goose isolate from Hungary, MYCAV93, forming a unique subclade among the *M. anserisalpingitidis* strains.

The antibiotic susceptibility profile of the isolated swan goose *M. anserisalpingitidis* strains also showed high similarity with the European isolates [20]. The quick development of multi-resistance of *M. anserisalpingitidis* against different antibiotics is regularly experienced in Europe, and it was confirmed by the comparison of the antibiotic susceptibility profile of *M. anserisalpingitidis* isolates from treated and non-treated birds from China also. Tylvalosin and tiamulin are the most potent antibiotics against mycoplasmosis in goose in both China and Europe. The elevated MIC values against a commensal bacterium, *M. cloacale* and the detected rapid development of resistance in *M. anserisalpingitidis* highlight that susceptibility test based targeted antibiotic therapy is strongly recommended in the geese farms.

## Conclusions

Our study highlights the importance of *M. anserisalpingitidis* infection in the goose industry on a global scale. The extensive/semi-intensive production system of goose hamper the eradication of the agent from a farm thus antibiotic therapy is the primary option of disease control. Unfortunately it not only means disease treatment but often prophylactic antibiotic application as well which quickly ends up in diverse antimicrobial resistance in *M. anserisalpingitidis* and probably in other bacteria colonizing the goose including zoonotic agents. Thus the development of a vaccine, as a long-term disease control option has to be considered.

## Methods

Samples were collected from a swan goose breeding farm with 75,000 animals 300 km southwest from Guanghzou in China in September 2019. A total of 12 cloaca swabs were collected from eight male (seven birds showing cloaca and phallus inflammation and one clinically healthy gander) and four female (all clinically healthy) swan geese, from live animals (Table 1). According to the written declaration (reference number: IVMR/2019/0023) of the Ethics Committee of the Institute for Veterinary Medical Research, Centre for Agricultural Research ethical approval was not required for the study as the samples were taken during routine diagnostic examinations with the written consent of the owner. The swabs were put on FTA cards (Whatman, Sigma-Aldrich Inc., Germany), first for future DNA extraction and then were also put in 2 ml of *Mycoplasma* broth medium (pH 7.8) (ThermoFisher Scientific Inc./Oxoid Inc./, Waltham, MA) supplemented with 0.5% (w/v) sodium pyruvate, 0.5% (w/v) glucose and 0.005% (w/v) phenol red, 0.15% L-arginine hydrochloride (w/v), filtered through a 0.65 μm pore size syringe filter (Sartorius GmbH, Goettingen, Germany), transported to the laboratory (1 week) and incubated at 37 °C. The cultures were inoculated onto solid *Mycoplasma* media (Thermo Fisher Scientific Inc./Oxoid Inc./) supplemented with 0.15% L-arginine hydrochloride (w/v) after color change of the broth and were incubated at 37 °C and 5% CO_2_ until visible colonies appeared (1–2 days). Purification of mixed cultures was performed by one-time filter cloning, minimizing the in vitro adaptation and mutations of the isolates. The QIAamp DNA Mini Kit (Qiagen Inc., Hilden, Germany) was used for DNA extraction according to the manufacturers’ instructions from the cultures and from the FTA cards. Species-specific PCR systems targeting *M. anserisalpingitidis, M. anatis, M. anseris, M. cloacale* and *Acholeplasma* species were used for screening and identification [21, 22]. The purity of the cultures was confirmed and the unknown species were identified by a universal *Mycoplasma* PCR system targeting the 16S/23S rRNA intergenic spacer region in *Mycoplasmatales* followed by sequencing on an ABI Prism 3100 automated DNA sequencer (Applied Biosystems, Foster City, CA), sequence analysis and BLAST search [23, 24].

Whole genome sequencing of an isolated strain (MYCAV785 from bird 12A) was performed on NextSeq 500 Illumina equipment (Illumina, Inc. San Diego, CA USA) using NextSeq 500/550 High Output Kit v2.5 reagent kit, resulting in 150 bp long single reads. The reads were quality checked with FastQC software version 0.11.8 [25]. A draft genome was assembled with the SPAdes software version 3.13.0 [26] and aligned with the three currently available *M. anserisalpingitidis* strains from the NCBI database (Accession number: CP042295.1 for ATCC:BAA-2147, CP041663.1 for MYCAV93 and CP041664.1 for MYCAV177). The alignment was performed with MAUVE version 20150226 [27] and the single nucleotide polymorphisms (SNP) found in the alignments were exported.

The Medusa web server [28] was used to create contigs from the previously assembled scaffolds. The longest contig was 0.941 Mbps similar in size to the other *M. anserisalpingitidis* whole genomes, as such this contig was chosen for use in further study. The genomes were also analyzed with BLAST Ring Image Generator (BRIG) [14].

The 16S rRNA sequence of *M. anserisalpingitidis* strain MYCAV785 was compared with other *Mycoplasma* species in the Synoviae cluster [8, 29] with *M. cloacale* strain 383 (GenBank accession number: NR_024985.1) chosen as outgroup. The genetic sequences were aligned with MAFFT algorithm [30] with the Unipro Ugene software version 33.0 [31]. A Maximum likelihood phylogenetic tree was constructed with the MEGA X software version 10.1.5 [16] using Generalised Time Reversible model with Gamma distribution with invariant sites (GTR + G + I) and 1000 bootstrap.

The sequence similarity of a 1997 bp fragment of the *rpoB* gene of strain MYCAV785 was examined with NCBI BLASTn search tool [24, 29]. Sequence identity was evaluated when query cover was 100%.

The following antimicrobial agents were examined during the microbroth dilution tests: the fluoroquinolones: enrofloxacin (batch SZBA336XV) and difloxacin (SZBD178XV); the aminocyclitol: spectinomycin (batch SZBB166XV); the lincosamide: lincomycin (batch SZBC340XV); the tetracyclines: doxycycline (batch SZBD007XV), oxytetracycline (batch SZBC320XV) and chlortetracycline (batch BCBR8687V); the macrolides: tilmicosin (batch SZBC345XV) and tylosin (batch SZBB160XV); the pleuromutilin: tiamulin (batch SZBC026XV); and the phenicol: florfenicol (batch SZBC223XV); all products originated from VETRANAL, Sigma-Aldrich Inc. The macrolide tylvalosin (Aivlosin, ECO Animal Health Ltd., UK; LOT M102A) was also included in the examinations. Lincomycin and spectinomycin were applied also in combination at a ratio of 1:2. Twofold dilutions were prepared in the range of 0.039–10 μg/ml for fluoroquinolones, doxycycline and tiamulin, 0.25–64 μg/ml for spectinomycin, lincomycin, lincomycin-spectinomycin (1:2) combination, oxytetracycline, chlortetracycline and macrolides and 0.125–32 μg/ml for florfenicol.

The microbroth dilution examinations on 10^4^–10^5^ (color changing unit) CCU/ml of the strains were performed according to Hannan [32]. The strains were tested in duplicates and the plate contained a duplicate of the *M. anserisalpinigitidis* type strain (ATCC BAA-2147) as a quality control. MIC was established as the lowest antibiotic concentration where no color change of the broth was observed as a consequence of the absence of bacterial metabolism. MIC values were recorded when color change of the broth media of the growth control was visible (initial MIC).

## Supplementary information

**Additional file 1.**

## Data Availability

All data generated or analysed during this study are included in this published article. The sequence reads of strain MYCAV785 gained by sequencing on NextSeq 500 Illumina equipment have been uploaded to the SRA under Bioproject number PRJNA602206. GenBank accession numbers of the 16S rRNA and *rpoB* gene fragments of MYCAV785 are MT241511 and MT241512, respectively (Supplement material).

## Abbreviations

BRIG: BLAST ring image generator
CCU: Color changing unit
SNP: Single nucleotide polymorphism
SRA: Sequence read archive
