# Supplementary material for: Isolation of Mycoplasma anserisalpingitidis from swan goose (Anser cygnoides) in China
Source: BMC Vet Res. 2020 Jun 5;16:178. doi: 10.1186/s12917-020-02393-5 (PMC7275505; doi:10.1186/s12917-020-02393-5)
Supplement: Supplementary file 1 — Additional file 1. [file 12917_2020_2393_MOESM1_ESM.pdf]

#MYCAV785_16S_rRNA_(MT241511):

ACATGACAGTCAATTTTTTCGAGAGTTTGATCCTGGCTCAGGATGAACGCTGGCTGTGTGCCTAATACATGCAT
GTCGAGCGGAGTTCTTCGGAACTTAGCGGCGAATGGGTGAGTAACACGTACTTAACATGCCCTCTAGATTGGA
ATAACGATTAGAAATGATCGCTAATGCCGGATACTTATATTTTTCGCATGAAGAATATATAAAAGGAGCGTTTG
CTTCGCTAGAGGATTGGGGTGCGTAACATTAGCTAGTTGGTAGGGTAATGGCCTACCAAGGCTATGATGTTTA
GCGGGGTTGAGAGACTGATCCGCCACACTGGGACTGAGATACGGCCCAGACTCCTACGGGAGGCAGCAGTA
GGGAATTTTCCACAATGGACGAAAGTCTGATGGAGCGACACAGCGTGCAGGATGACGGCCTTCGGGTTGTAA
ACTGCTGTTATAAGGGAAGAAAAAATAAAGTAGGGAATGACTTTATCTTGACGGTACCTTGTCAGAAAGCAAC
GGCTAACTATGTGCCAGCAGCCGCGGTAATACATAGGTTGCAAGCGTTATCCGGAATTATTGGGCGTAAAGCG
TCTGTAGGTTGTTTGTTAAGTCTGACGTGAAAACTTGGGGCTCAACCCCAAATTGCGTTGGATACTGGCAAACT
AGAGTTATGAAGAGGTTAGCGGAATTCCTTGTGAAGCGGTGAAATGCGTAGATATAAGGAAGAACACCAACT
TGGCGAAGGCAGCTAACTGGGCATACACTGACACTGAGAGACGAAAGCGTGGGGAGCAAACAGGATTAGAT
ACCCTGGTAGTCCACGCTGTAAACGATGATGATTAGCTGATGGGAAACTCATCGGCACAGCTAACGCATTAAA
TCATCCGCCTGAGTAGTATGCTCGCAAGAGTGAAACTTAAAGGAATTGACGGGGATCCGCACAAGCGGTGGA
GCATGTGGTTTAATTTGAAGATACGCGTAGAACCTTACCCACTCTTGACATCTTCCGCAAAGCTATAGAGATAT
AGTGGAGGCTAACGGAATGACAGATGGTGCATGGTTGTCGTCAGCTCGTGTCGTGAGATGTTCGGTTAAGTCC
TGCAACGAGCGCAACCCTTGTCCTTAGTTAAATGTTCTAAGGAGACTGCCCGAGTAATTGGGAGGAAGGTGG
GGACGACGTCAAATCATCATGCCTCTTACGAGTGGGGCAACACACGTGCTACAATGGACGGTACAAAGAGAC
GCAATACGGCGACGTGGAGCAAATCTCAAAAAACCGTTCTCAGTTCGGATTGTAGTCTGCAACTCGACTACAT
GAAGTCGGAATCGCTAGTAATCGTAGATCAGCTACGCTACGGTGAATACGTTCTCGGGTCTTGTACACACCGC
CCGTCACACCATGGGAGCTGGTAATGCCCGAAGTCGGTTTTGTTAACTACGGAGACAACTGCCTAAGGCAGGA
CTGGTGACTGGGGTGAAGTCGTAACAAGGTATCCCTACGAGAACGTGGGGATGGATTACCTCCTTTCTA

#MYCAV785_-_DNA-directed_RNA_polymerase_subunit_beta_(rpoB)_CDS_(MT241512):

ATGGATCAAAATTACAGAATTAGTACATTCGGAAAAAATACTCAAAGAAGAGATTACTCAGTAACTAAGCATT
CTCTTCCAGTTTTTAATATCTTATCAACTAGCAAAGATAGTTTTGATAATTTCTTAAAAAATAACATCGAAGAGG
CTTTATTAGAACATTATCCAATTGAAGCCGCTGACAAAAAAGTTAAACTTGATTATGTAAAGAAAAGTTTAAGA
ATTGCATATCCTTACAAAAAAAATTACTAGTGAAGGTGAAGAAATTAGAAAATGTAAAGCTAAAGGGATAAATT
TCTCTGCTAAAGTTTATGCAACTTTAAAGCGTGAAATTACAAGTACAGGAGAAGTTAAAGAAGATGAAGTTCTT
CTTGGAGAAATCCCTTTAATGACAAGTGGCGGAAGCTTTATCATTAACGGTTCTGAGAAAGTTGTTGTTAGTCA
GTTGATTAGATCGCCTGGTGCGTACTTCGGAAGAGGTGTTCGTAACAAGCAATCTGATGACCTTTTTAACAAAT
TAGAAATTTTACCTAGAATTGGATCATGAGTTGAAGTTTCACACAAAATTACTTCGTCTAATGTTGACACAGTA
AAAATTAAAATAGACAAAAATAAAAACGTAGCTTTATCAACCTTCTTAGTGGCTTTAGGTATTATGCCTGAAAC
AATTAAAAAATTATTCGGAACAAGTGAAGAATTGTTAGAAACACTTAATAGAGATAAGTTAATTAATACACCTG
AAATCAGTGATGAAGAAATGATTGATATGTGTCAAGAAGAATTATTCAGATGATTAAGAAAAGGTGACCGTAT
TTCTGTTGATGCTAAAAAATCATTAATTCCAAACATTTTATTTGATAAGAAACGTTACAACCTTTCTGCAACAGG
TCGTTATATGTTAAACAACAAATTGAGTGTTGTTGACCGTATTGTTGGAACATATTTAGCTGAAGATCTTAAAG
CTAAAAGTGGTGAAGTGTTATTCACTAAAGGAACACTTATTACACATGAAATAGCTTTAGAAATTAATAAATCA
TTCAAAGAGAAAATTTTAGCTATGGATTATATTGAAAATGTTAATCCAGAATTGGTTTACTATAACTTAATCACT
CCTCAAACTAAGAGTTTAGCTAAAAGAATTAAAATTTCTAAAGTCAAAGTATACCCAAATAGAAAAAGTTTAGA
AGATGAAAATATTGAACCAATTTTAGTTATTGGTAATGATCAACGTTCTGATGAACAACATTTATTAATTTCTGA
TATTGTTGCTGCCATAAGCTACTACTTTAACTTAATTGATGGAATAGGTACTGATGATGATCCTGACTCAATGGT
AAACAAAAGAATTGTTTCTGTAGGAGAATTATTAGCTAATCAATTAAATGTAGCATTATCAAAATTGGAAAAAA
CTACGCGTGAAAGAATGGGAGCTAAAGAACCTGAAAAAGTAACAGCGAAAAATGTTACAAATAATAAATTGA

TCACAAATCAAATGAAAACATTCTTTAACTCATCAAAACTTTCTCAATTCATGGACCAAATTAACCCTCTAGCTG
AAGTGTCGAATAAACGTCGTGTTACTTCTCTTGGGCCTGGTGGTCTTAACCGTGATACTGCTCAATTCGAAGTT
CGTGACGTTCATTCAACTCACTATGGAAGAATTTGTCCTATCGAAACCCCAGAAGGACCAAACATCGGACTTAT
CTTAAACTATGCAACATATGCAAAAGTAAATGAAATGGGATTCTTACAAACTCCTTATTTCAAAGTTAATAATG
GTGTTATTGATTACAATGATGTTAGATATTTAACAGCTTCTGAAGAAATTGGATATAAATTCTCTCAATCATCAG
TAACTGTTGATGAAAACAATAGAATTGTTGATGAATTAATCACAATTAGACATAACTATAACTATGTTATTGATA
AACCAAGTGAAGTAGATTTTATTGAGGTTTCATCAAAACAAATGGTTTCGGTAGCTGCTGGTGCAATTCCTTTC
TTAGAAAATGACGACGCTAACCGTGCCCTTATGGGTTCTAACATGCAACGTCAAGCAGTTCCTCTTCTTCAAGC
TGAAGCTCCTTATGTTGCAACAGGTATTGAAGGAGACATTGCTAAATATTCAGCATACAACTTAGTAGCTAAAA
ATGCTGGTGAGGTTGTTTATGTTGACTCACAAAAAATTCACATTAAAAATGATAAAGGAACAACTGATAAATAT
GTTTTAAGAAACTTTGAAAGATCAAATCAAGGTACAGTTATTAACCAAATTCCAATTGTAAAACTTGGTGATAA
AGTTGAAGAAGGCGAATTAATCGTTGATGGTTCATCATTCAAAAATGGAGAATTAGCTTTAGGTAAAAACGTT
TTAGTTGCTTTTACAACTTGAAACGGATATAATTACGAAGATGCTATCATCTTAAATGAAAAGCTTGTTAAGGA
TGATGTTTATACTTCTATTCATATTGAAGAACAAACAATTCAATTCAGAAATAGTAAGGCTGGTGATGATAAAT
TAACAGCTTCAATTCCAAACGTTTCAAAATATTCATTAAGAAACTTAGATGAAAATGGAATTGTTAGGGTTGGT
TCTGAAGTAGTTCCTGGTGATGTTCTAGTAGGTCGTGTAAGCCCTAAAGGTGAAGAAAATCCAACTCAAGAAG
AAAAATTATTAATGGCCATCCTTCAACAAAGACCTTCAAGTGATAGAGATACATCATTAAAAGTAAAAAATGGT
CACAACGGTACAGTTATAGGTGTTGAAGTATTAAGTAGAGAACTTGGTGATGTTCTTGAAGATGGTATTGACA
AGATTGTTAAAGTTTCTATTGCTCAAAAGAGAAAAATTAAAGTTGGTGATAAGATGGCTGGACGTCATGGTAA
CAAAGGGGTTATCTCTATTGTTTTACCTGAAGAAGATATGCCACACCTTGAAGATGGTACACCAGTAGATATCA
TGCTTAACCCTCAAGGGGTTCCATCGCGTATGAATATTGGTCAAGTTCTTGAACTTCACCTTGGAATGGCTGCG
CGTAAACTTGGTGTAAAATTCGTTACCCCTTCATTTGATGGTGTTAAAAAACAAGATATCGAAAACGCTCTTGA
AGAAGCTGGACTTCCAAAAACAGGAAAACAAATTTTAATTGATCCAGTTACAGGTGAAAAATTTGACAACCCA
GTTTCTGTTGGAGTTATGTACATGCTCAAACTTAACCACATGATCGACGATAAAATGCACGGTCGTAGTGTTGG
TCCATACTCATTAATTACACAACAACCACTTGGAGGAAAAAGTCAAAACGGGGGTCAAAGATTTGGAGAAATG
GAAACATGAGCTATTGAAGCTTATGGTGCAACAAATGTTCTACAAGAAATCTTGACTTACAAATCTGATGATAT
TCAAGGAAGAAACTTACTTTATAGTGCTTTAGCTAGTGGTAGAGAATTGCCTAAACCAGGTACACCTGAATCAT
TTAATGTTCTTAGCTATGAACTTAGAGGTTTAGGAATGAAATTAACTCTTAAAGAGAAAAATAACGAGGAAGA
AGATGATGATATCTTCCAATACCTAGAATCAGGAGAGGTAGATAATGAATAA
